# Physiological Assessment of Mental Stress in Construction Workers Under High-Risk Working Conditions: ECG-Based Field Measurements on Inexperienced Scaffolders

**DOI:** 10.3390/s26030949

**Published:** 2026-02-02

**Authors:** Likai Lei, Shiyi He, Ruihao Hou, Yifan Zhu, Jiaqi Zhao, Yewei Ouyang

**Affiliations:** 1 Sino-Australia Joint Research Center in BIM and Smart Construction, Shenzhen University, Shenzhen 518060, China; leilikai2025@mails.szu.edu.cn; 2 College of Civil and Transportation Engineering, Shenzhen University, Shenzhen 518060, China; 3 Key Laboratory of Physical Fitness and Exercise Rehabilitation of Hunan Province, Hunan Normal University, Changsha 410081, China; heshiyi@hunnu.edu.cn (S.H.);; 4 Engineering Digital Technology R&D Center, Engineering Design & Research Institute of CCCC THEC, Beijing 100102, China; zhuyf12@foxmail.com

**Keywords:** mental stress, HRV, inexperienced scaffolders, working at height

## Abstract

High-risk working conditions in construction, such as working at height, may elicit elevated mental stress in workers and pose significant safety challenges. This study aims to physiologically assess construction workers’ mental stress under high-risk working conditions using heart rate variability (HRV) features derived from electrocardiograph (ECG) signals. An experimental study in the field was conducted, where inexperienced scaffolding workers’ (*n* = 20) ECG signals were collected when working at three different heights corresponding to low, medium, and high levels of mental stress. Supervised machine learning algorithms, including Support Vector Machine (SVM), KNearest Neighbor (KNN), Linear Discriminant Analysis (LDA), and Random Forest (RF), were applied for model development. The results show that the HRV features obtained good prediction accuracy. The classification accuracy is up to 85.00% between low and medium stress levels, 92.50% for differentiating low and high stress levels, and 87.50% for classifying medium and high stress levels. These findings demonstrate the potential of ECG-derived HRV features for differentiating the mental stress responses of construction workers under high-risk working conditions and provide empirical evidence supporting the feasibility of physiological monitoring of workers’ mental stress in real construction environments.

## 1. Introduction

Mental stress refers to the body’s response to a psychological barrier (e.g., excessive job demands) [1]. Construction workers frequently suffer from mental stress [2]. There are many job stressors in construction workplaces; e.g., workers may suffer chronic stress due to poor support from colleagues and conflicts on the job [3] and management issues (e.g., working overtime and unfair management treatment) [4,5], and they may also have immediate stress due to working in a hazardous environment [6].

High-risk working conditions in construction, particularly tasks involving work at height, represent a prominent source of acute mental stress. When working under such conditions, workers are exposed to an elevated risk of falls and severe injuries, and perceived unsafe working environments have been identified as a major contributor to psychological stress [7,8]. Mental stress experienced under high-risk conditions has been associated with adverse psychological and physiological symptoms [9], increased proneness to errors and mistakes [10], and a reduced likelihood of engaging in safe work behaviors [3], ultimately increasing the probability of safety incidents.

The timely identification and monitoring of workers’ mental stress are therefore critical for proactive safety management in construction. The real-time assessment of mental stress can help identify workers at elevated risk and support decision-makers in implementing targeted interventions. Compared with subjective or behavioral approaches, physiological measurement has been widely recognized as a promising method for mental stress assessment due to its objectivity, non-intrusiveness, and ability to provide continuous information with minimal interference to task execution [11]. Past studies have investigated electroencephalography (EEG) [12], heartbeat features, skin temperature, breathing rate [13], and skin conductance [14] in estimating the mental stress of construction workers. However, many of these approaches face practical challenges for on-site application, such as movement constraints, susceptibility to environmental noise, or the complexity of multi-sensor configurations. Moreover, existing evidence is largely based on generic hazardous tasks or laboratory-like settings, and empirical research focusing specifically on mental stress during real working-at-height scenarios remains limited. It therefore remains unclear whether existing physiological stress assessment models can be directly transferred to such high-risk construction contexts.

To address these gaps, this study investigates the potential of heart rate variability (HRV) features derived from electrocardiogram (ECG) signals for assessing mental stress in construction workers performing tasks at height. HRV reflects variations in the intervals between successive heartbeats and is influenced by autonomic nervous system regulation [15]. Because ECG signals can be collected using wearable and relatively unobtrusive devices (e.g., chest straps or wearable sensors) [16], HRV-based assessment offers practical advantages for field-based monitoring in construction environments.

Accordingly, this study examines the potential of ECG-derived HRV features to assess the mental stress responses of inexperienced scaffolders performing tasks under high-risk working conditions. By developing HRV-based stress classification models using field-collected data, this research aims to provide empirical evidence on the feasibility of the physiological assessment of mental stress in construction settings and to improve the understanding of the relationships between workers’ physiological responses and mental stress under high-risk operational contexts.

## 2. Related Works

### 2.1. Mental Stress Measurement in the Construction Field

Methods for measuring mental stress include subjective, behavioral, and physiological measures [17]. Subjective self-reports and standardized questionnaires (e.g., PSS, STAI) are commonly used [18], but their applicability in construction settings is limited by recall bias, reporting burden, and the difficulty of frequent or continuous assessment during ongoing work. Behavioral paradigms such as task-based stress tests (e.g., TSST, MIST) can provide controlled stress induction [19,20], but typically require interrupting workers’ primary tasks, and are therefore less suitable for field-based monitoring.

In contrast, physiological approaches offer a more objective and continuous means of stress assessment and have attracted increasing attention in construction research [14,21]. Among these, EEG-based methods have demonstrated promising performance in controlled or semi-controlled construction-related scenarios [12,22]. However, their practical application on active construction sites remains challenging due to equipment intrusiveness, susceptibility to environmental noise, and operational constraints [21,22].

As a result, recent studies have increasingly explored peripheral physiological signals, such as cardiovascular indicators, respiration, skin temperature, and electrodermal activity, for stress monitoring in construction contexts [13,14]. These studies have reported encouraging classification performance; e.g., one study [13] achieved an accuracy of 94.7%. Nevertheless, many of them rely on multi-sensor configurations, which can increase data collection complexity and reduce feasibility in real-world environments [11,21].

Moreover, existing empirical evidence is largely based on cognitive stress tasks or specific scenarios such as ladder climbing or confined spaces, rather than genuine working-at-height conditions in real construction settings [12,13]. Given that mental stress induced by different stressors (e.g., cognitive load, time pressure, perceived physical danger) may involve distinct physiological patterns, the transferability of existing models to high-risk working-at-height scenarios remains uncertain [22]. To date, empirical evidence on physiological stress responses of construction workers performing real tasks at height is still limited [21].

### 2.2. Potential of HRV in Mental Stress Measurement

HRV has been demonstrated to be impacted by mental stress, which supports its use for the objective assessment of mental stress [23]. Several HRV features are associated with mental stress. For example, the decreased values of the mean of RR intervals (the time gap between two consecutive normal R peaks of the ECG signal) indicate that people’s resting heart homeostasis moves to the sympathetic side under stressful states [24].

HRV has also been demonstrated as a potential biomarker for the practical monitoring of mental stress [25], showing good performance in estimating mental stress in other fields. The researchers in [26] induced participants’ mental stress with a Stroop color-word test and a math test. They reported that average stress classification accuracy for two-level and three-level stress could achieve 96.7% and 75.6% by applying twenty HRV features. Study [27] examined the absolute reliability, relative reliability, and statistical significance in differentiating the low and high stress levels of 42 HRV metrics. The results showed that 22 metrics show absolute reliability, 21 show relative reliability, and 13 differentiate low and high stress levels.

Applying HRV in construction workplaces also has some advantages. First, with fast-developing unobtrusive sensors such as wristbands, armbands, chest straps, and embedded sensors, data could be collected through these wearable sensors with little interference to workers at job sites [16]. Second, applying one single sensor could reduce the difficulty of data collection and processing compared to using multiple sensors. These characteristics make HRV a particularly suitable candidate for the physiological assessment of mental stress in high-risk construction scenarios, where both worker safety and operational continuity are critical.

## 3. Methodology

**Figure 1:**
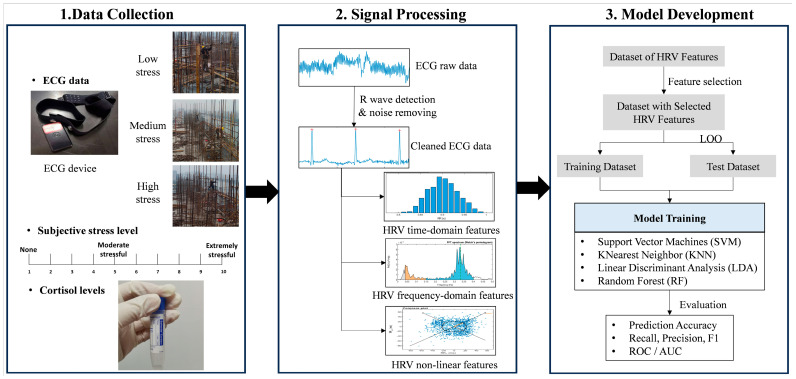
Research framework.

As shown in Figure 1, ECG data were collected from workers while they performed tasks under different working-height conditions on site. Subjective stress ratings and cortisol levels were obtained concurrently to support the differentiation of workers’ mental stress responses across conditions. The physiological raw data were subsequently preprocessed to obtain cleaned ECG signals, from which HRV features were extracted as inputs for stress classification models. Machine learning methods were then applied to develop HRV-based models for differentiating workers’ mental stress responses under high-risk working conditions.

### 3.1. Subjects and Apparatus

A total of 20 male construction workers joined the study. The workers were inexperienced scaffolders with less than one year of work experience (3 subjects with less than 3 months’ experience, 4 with less than 6 months’ experience, 7 with less than 9 months’ experience, and 6 with less than 12 months’ experience). The subjects with less than one year of working experience were invited because they are regarded to be exposed to higher risks due to lack of experience [28]. The participants were aged between 19 and 23 (mean = 21.15; SD = 1.28). In addition, all participants were screened to ensure they had no known history of cardiovascular, respiratory, neurological, or endocrinological diseases that could potentially affect heart rate variability (HRV). The participants were also instructed to avoid alcohol, caffeine, and strenuous physical activity prior to the experiment to minimize potential confounding effects on physiological measurements. The Ethical Research Committee at the Hunan Normal University approved the study (NO.370). Informed written consent was obtained from each participant.

ECG recordings were obtained using a Custo Guard Holter system (Custo Med GmbH, Ottobrunn, Germany), with one electrode positioned at the fourth intercostal space to the right of the sternum. The ECG sensor recorded data at a frequency rate of 512 HZ.

### 3.2. Data Collection and Preprocessing

The workers were required to work at three different working heights: one was on the ground, and the other two were set heights above the ground, with a lower and a higher working surface. The three working-height conditions were designed to represent different levels of perceived risk. Workers’ mental stress responses under these conditions were empirically examined and supported using subjective stress ratings and cortisol measurements.

The ground-level condition was designed to represent a relatively low-stress working scenario. Before data collection, participants were given a brief adaptation period to familiarize themselves with the experimental setting and equipment. During the ground-level measurement, participants performed routine tasks under stable conditions, without time pressure or hazardous exposure.

Moreover, because of possible differences in sensitivity to working heights between individuals, the two working heights for off-ground work were determined on an individual basis instead of being standardized for each worker. Each worker was asked at what height he would start to feel moderate and high stress, respectively. This individualized height-setting approach was adopted to better capture workers’ perceived risk thresholds.

This study was carried out on the steel pipe scaffolding of residential buildings. The lowest working height above the ground was about 1.8 m, i.e., one step length of the scaffold. The highest distance above the ground was less than 20 m because there was a platform about every 20 m. HRV measurements were taken continuously for 1 h from the start of work at height. All measurements for each participant were conducted within the same time window of the day across the three working-height conditions to minimize intra-individual time effects. Although the exact testing time varied between participants due to field constraints, data collection was generally scheduled within a relatively consistent daytime period to reduce the influence of circadian variation. To minimize potential order effects, the order of the three conditions was arranged using a Latin square design across participants.

To examine the differentiation of stress responses across working-height conditions, their subjective reports about their stress levels and their cortisol levels were measured. The subjects reported their level of mental stress while performing the task immediately after finishing it (i.e., How much pressure did you feel to finish the experimental task?), using a 0–10 scale, with 0 indicating no stress and 10 indicating maximum stress, and higher values indicating more stress. In addition, the workers’ cortisol levels were measured while working under the different conditions. This single-item scale was adopted to minimize participant burden and disruption in the field-based, high-risk experimental setting and to enable rapid assessment immediately after task completion. While multi-item validated questionnaires may provide more comprehensive psychometric assessment, their practical applicability in on-site construction environments is limited. Cortisol is known as a stress hormone and is highly associated with an individual’s stress [29]. Workers’ saliva samples were collected in the first ten minutes of working at height to test their cortisol levels. Specifically, the cortisol from the saliva samples was collected using salivettes (Sarstedt AG & Co., Nümbrecht, Germany). The participants were not permitted to eat, drink, brush their teeth, or take medicines for about an hour and were asked to rinse their mouths with water ten minutes before the experiment. A synthetic fiber roll was placed in the participants’ mouths for about 1 min (until saturated with saliva). After 2 min of 1000 g centrifugation, the supernatants were stored at 4 °C and then sent to a laboratory (Shanghai, China) for standard cortisol level analysis.

Kubios HRV Premium version 3.5.0 [30] was applied to process the raw ECG signals to remove noise. The R-wave time instants were detected by applying the QRS detection algorithm—Pan–Tompkins algorithm [31]. Band-pass filtering was used to reduce power line noise, baseline wander, and other noise components [30]. The automatic noise detection algorithm in Kubios was also utilized to automatically identify noise segments based on the raw ECG data. HRV analysis was conducted using the default Kubios processing settings unless otherwise specified. Artifact correction was performed using the Kubios automatic correction algorithm with the correction level set to medium. ECG was continuously recorded during task execution, and only the segments corresponding to active work performance were included in the analysis. The 1 h ECG recording for each condition was segmented into consecutive 5 min windows, and HRV features were extracted from each window. Frequency domain indices were computed using the Welch power spectral density method. The standard frequency bands were defined as very low frequency (VLF) (0.0033–0.04 Hz), low frequency (LF) (0.04–0.15 Hz), and high frequency (HF) (0.15–0.40 Hz).

### 3.3. HRV Feature Computation

Based on comprehensive literature reviews [25] and original research articles [26,32], this study selected 14 HRV features listed in Table 1 that might have potential in classifying mental stress. The time domain indicators, including the mRR, SDNN, RMSSD, and pNN50, which could indicate changes of the sympathetic nervous system (SNS) and parasympathetic nervous system (PNS), and metrics about heart rate (mHR) were calculated. RR interval is the time gap between two consecutive normal R peaks of the ECG signal. Both SNS and PNS activity contribute to SDNN. The RMSSD reflects the beat-to-beat variance in HR and is the primary time domain measure used to estimate the vagally mediated changes reflected in HRV [15]. The pNN50 is closely correlated with PNS activity [15]. Frequency domain HRV metrics complement time domain metrics in offering extra information about the properties of oscillatory components in the heart rate dynamics. Three main spectral components (VLF, LF, and HF) and total power (TP) (a measure of overall autonomic activity) were analyzed. PNS activity may contribute to VLF power, LF power may be produced by both the PNS and SNS, and the HF band reflects PNS activity [15]. Except for the absolute power (ms^2^), LF and HF were also measured in normalized units (n.u.). In addition, the LF/HF ratio, which can be interpreted as the balance between parasympathetic and sympathetic activity [33], was also calculated. Regarding non-linear features, Poincaré plot analyses were added for mental stress classification. A Poincaré plot is graphed by plotting every R–R interval against the prior interval, creating a scatter plot. It allows for visually searching for patterns buried within a time series. SD1 and SD2 refer to the standard deviation perpendicular to and along the line of identity. SD1 correlates with baroreflex sensitivity (BRS), and SD2 correlates with LF power and BRS.

### 3.4. Classification Model Development

Supervised learning algorithms were applied to develop stress classification models because they have been demonstrated to support a quick, accurate, and robust cognitive state detection by learning from individual physiological features [34]. Three classifiers were developed to differentiate the three stress levels. Figure 1 shows the procedures of classification model development.

Feature selection was performed to simplify the model structure, enhance interpretability, reduce the risk of overfitting, and lower computational burden during model training. Statistical analysis was used to identify features that were most relevant for distinguishing between the two classes. The paired *t*-test was used to rank the importance of the features according to the absolute values of t. As the difference between the sample data and the null hypothesis increases, the absolute value of the t-value increases [35]. The absolute value of the t-statistic was used as an indicator of each feature’s discriminative ability, with larger absolute values indicating stronger differentiation between the two sample groups. All features were initially included in model training, after which features were iteratively removed in ascending order of their absolute t-values. Features with smaller absolute t-values were eliminated first because they contributed less to class separation. The paired comparisons were intended as exploratory analyses to characterize discriminability patterns across stress levels rather than as confirmatory hypothesis testing. After feature selection, Min–Max normalization was applied to rescale all features to the range [0, 1], thereby reducing the influence of differences in feature scale.

Since 20 subjects is a small sample size for machine learning, overfitting may occur during model training. To prevent overfitting, cross-validation was used to improve the generalization of the model. Specifically, the leave-one-out cross-validation (LOO) approach was applied to split the training and test datasets. LOO partitions the data of one subject as the test dataset and the remaining subjects as the training dataset, enabling it to fully use each subject’s sample to reduce the subject bias caused by individual differences [36]. LOO can be especially useful for small sample sizes, and it can help to prevent overfitting by providing an estimate of the model’s performance on new data [37]. In addition, grid search was used for hyperparameter optimization, which gradually narrows the search range of hyperparameters and reduces the grid search step to find the optimal hyperparameters.

Four supervised learning algorithms were selected for training the models: Support Vector Machine (SVM), KNearest Neighbor (KNN), Linear Discriminant Analysis (LDA), and Random Forest (RF). SVM creates hyperplanes that separate data points of a binary classification problem. SVM is suitable for high-dimensional non-Gaussian distribution data, and many psychophysiological data have these characteristics [38]. Linear and RBF kernels will be tested in this study, and the hyperparameters C and γ are both defined in the range [2,3,4,5,25]. KNN uses the entire database for prediction based on a similarity measure in the instance space [39]. KNN is also robust to noisy data and has a low computational cost, making it a promising approach for industrial applications [34]. The hyperparameter K in this study is defined in the range [1,25]. LDA estimates separating hyperplanes by seeking the direction in feature space where projections of classes have the greatest inner-means distance and the smallest variance [40]. It is simple and has been used successfully in many Brain–Computer Interfaces (BCIs) [41]. The RF classifier yields reliable classifications using predictions from an ensemble of decision trees [42], which is demonstrated to be robust to noise in ECG data [43]. The Ntress is defined from 10 to 1000 trees in steps of 100, and Mtry is defined from 2 to the largest number of the features.

Finally, the classification performance of the models was evaluated using several standard metrics, including overall accuracy, recall, precision, F1-score, and the area under the receiver operating characteristic curve (AUC). In this study, samples corresponding to higher stress levels were defined as the positive class. Recall was calculated as the proportion of true positive samples among all actual positive samples, reflecting the model’s ability to correctly identify workers experiencing higher stress. Given that the primary objective of the models is to detect high-stress states, recall was considered a particularly important evaluation metric. Precision was defined as the proportion of true positive samples among all samples predicted as positive, indicating the reliability of positive classifications. The F1-score, defined as the harmonic mean of precision and recall, was used to provide a balanced assessment of model performance. The AUC was used as a threshold-independent indicator to compare the performance of different classification algorithms and feature sets, with larger values indicating better discriminative ability. All classification models and performance evaluations were implemented using Python 3.7.

## 4. Results

### 4.1. Validation of Stress Level Differences Across Experimental Conditions

To verify that the three experimental working-height conditions (ground-level, lower height, and higher height) corresponded to different levels of stress, subjective ratings and cortisol levels were analyzed as independent validation measures to confirm that the experimental conditions were associated with significantly different stress responses.

When completing the work on the ground, subjects indicated little stress, with choices centered on 0 or 1 (0.55 ± 0.51). Subjects responded with increased stress when they worked at height. At the lower height, they chose stress levels ranging from 3 to 6 (4.65 ± 1.04). At the higher height, they chose stress levels between 9 and 10 (9.55 ± 0.51). A one-way repeated measures ANOVA was conducted to compare the subjective stress levels among the three working conditions. The results show that the height of the working surface significantly influenced the subjective pressure ratings of the workers (*p* < 0.001). The pairwise comparisons show that the workers’ stress levels were also significantly different from one to the other: low and medium (*p* < 0.001), low and high (*p* < 0.001), and medium and high (*p* < 0.001). These results indicate a clear differentiation of subjective stress responses across working-height conditions.

**Figure 2:**
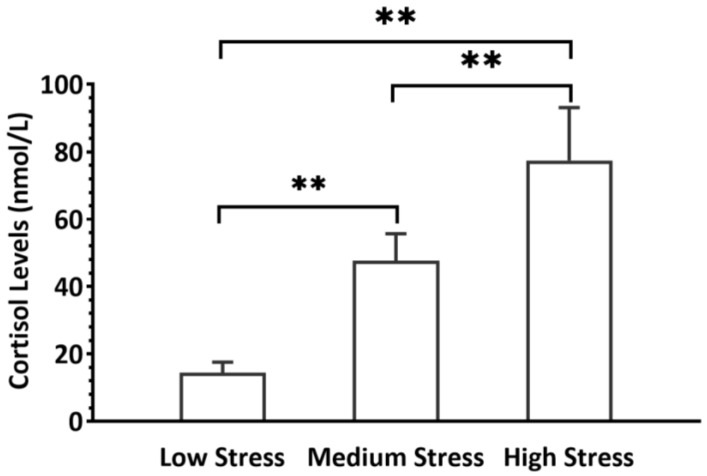
Results of cortisol levels (** significant level at 0.01).

Figure 2 shows the workers’ cortisol levels under the three working heights. Based on the results of the one-way repeated measures ANOVA, the height of the working surface significantly affected the stress levels of the workers (*p* < 0.001). There were also significant differences between every two stress levels. The results also supported the differentiation of workers’ mental stress responses across conditions.

### 4.2. Accuracy and Evaluation of Classification Models

**Figure 3:**
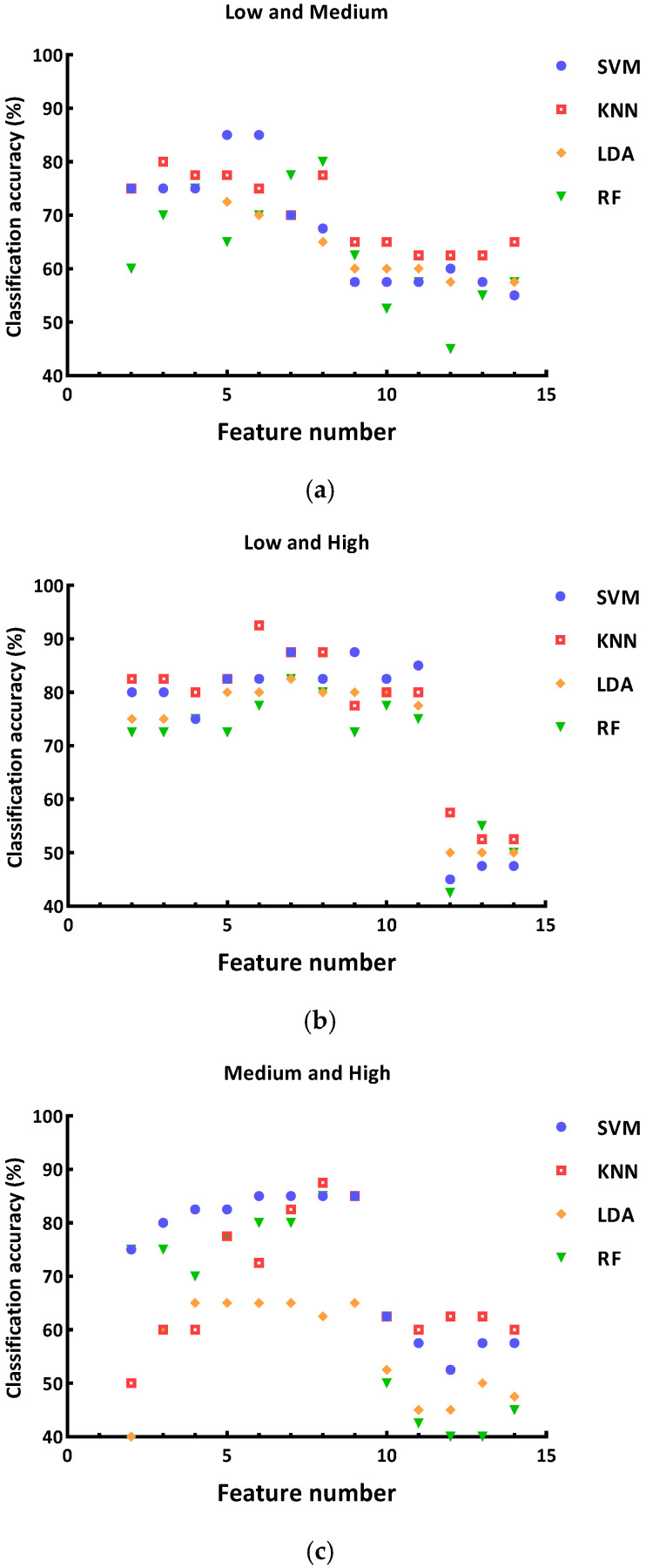
Summary of the classification accuracy: (**a**) classification accuracy between low and medium stress; (**b**) classification accuracy between low and high stress; (**c**) classification accuracy between medium and high stress.

The results of the paired *t*-test between two stressful states are shown in Table 2. The accuracy of the classification models was calculated when applying different numbers of features, as shown in Figure 3, where the combination of features was determined based on the value of t in Table 2. The absolute values of t were ranked in descending order, and the feature with the smallest absolute value was kicked out each time. As shown in Figure 3, when differentiating low and medium stress, applying six HRV features through the SVM algorithm (RBF kernels, C = 16, γ = 4) could achieve the best classification accuracy (85.00%). When differentiating low and high stress, applying six HRV features through the KNN algorithm (k = 5) could achieve the best classification accuracy (92.50%). When differentiating medium and high stress, applying eight HRV features through the KNN algorithm (k = 3) could achieve the best classification accuracy (87.50%).

**Figure 4:**
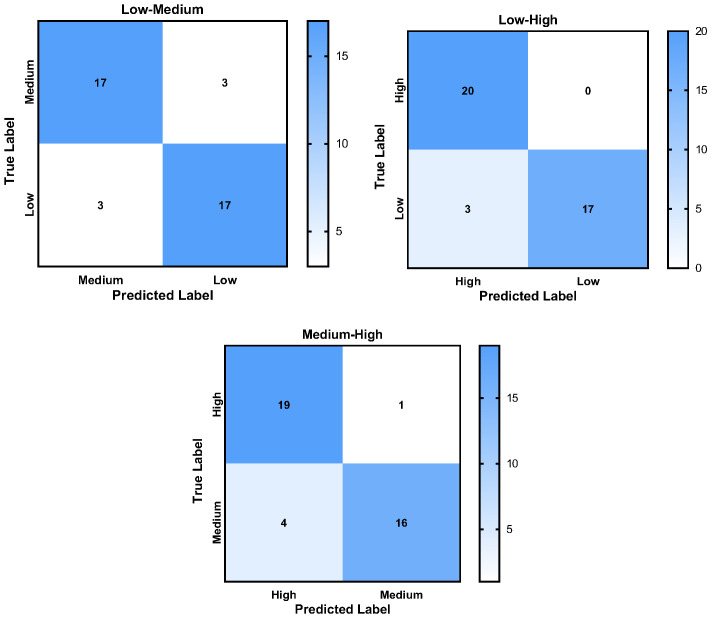
Confusion matrix of the classification models with the best classification accuracy.

**Figure 5:**
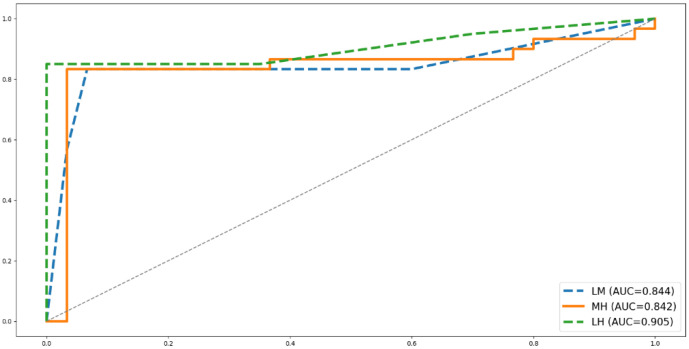
ROC curves of prediction models.

Table 3 shows the results of the evaluation metrics of the classification models with the highest accuracy, and the confusion matrix and ROC curves are respectively shown in Figure 4 and Figure 5. It shows that the model classifying low and high stress performed the best in all aspects. It not only has the highest classification accuracy, but also shows the largest recall, precision, F1, and AUC values. The model differentiating medium and high stress has a recall value of up to 0.95, indicating its ability to detect subjects with high stress.

## 5. Discussion

This study examined whether ECG-derived HRV features can be used to physiologically differentiate the mental stress responses of construction workers under high-risk working conditions. HRV features from three domains—time domain, frequency domain, and non-linear measures—were considered to capture different aspects of autonomic nervous system modulation associated with mental stress.

The time domain features, mRR, SDRR, and RMSSD, played key roles in classifying the mental stress levels. The results of subjects’ mRR and SDRR are in agreement with the meta-analysis [25], showing a decrease when people were stressed. The mRR could reflect the cardiac sympathetic to parasympathetic tone ratio [24]. The decreased values of mRR indicated that people’s resting heart homeostasis moves to the sympathetic side under stressful states. RMSSD is commonly regarded as an index of parasympathetic activity and has been shown to decrease under mental stress, reflecting vagal withdrawal [15]. These results suggest that time domain HRV features provide meaningful physiological indicators of stress-related autonomic modulation in high-risk construction contexts.

Frequency domain measures perform Fourier transforms on RR series, which could reflect sympathetic and parasympathetic activities using specific frequency bands. The results show that mental stress is very sensitive to frequency domain features, with over six frequency domains appearing in the feature combinations of the prediction models. This may be because the normal balance between the SNS and PNS may be altered under mental stress [44]. The autonomic nervous system (ANS) is divided into the SNS and PNS, which control emergency situations and relaxed activities. Frequency domain features can comprehensively indicate ANS activity. In the present study, a decrease in HF was observed under higher stress conditions. Such a pattern has been reported in previous stress-related studies and may reflect reduced parasympathetic modulation under stress exposure [33]. These findings further support the physiological plausibility of using HRV features to characterize mental stress responses in high-risk working environments.

Compared with previous construction safety studies that relied on multiple physiological sensors, the present study demonstrates the potential advantages of using HRV features derived from a single ECG sensor. Prior research has shown that integrating heart rate with additional signals such as skin temperature, breathing rate, and skin conductance can achieve high classification accuracy [13]; however, such multi-sensor approaches increase the complexity of data acquisition and may limit feasibility in real construction settings. Moreover, relying solely on heart rate without extracting HRV features may reduce sensitivity to mental stress, particularly for cognitively induced stress responses [13]. In contrast, the present findings indicate that a relatively small number of HRV features can effectively differentiate stress responses, enabling rapid signal processing and supporting the feasibility of low-cost, real-time physiological assessment on construction sites.

Supervised learning algorithms could utilize labeled training datasets to teach models to produce a desired output. Previous studies demonstrated that supervised machine learning could be a promising approach for performing cognitive state detection based on HRV features (e.g., [34,45]). Four algorithms were used in this study, where SVM and KNN showed superior performance. Similarly, many previous studies reported superiorities of SVM in detecting cognitive states based on psychophysiological measures [45,46]. This is probably because SVM is suitable for high-dimensional non-Gaussian distribution data, and many psychophysiological data have these characteristics [38]. However, SVM might have difficulty in training large-scale datasets [47]. In contrast, KNN was reported to be compatible with large-scale samples [48]. These characteristics suggest that different supervised algorithms may be appropriate depending on data scale and application requirements, and that HRV-based stress assessment can be flexibly implemented in industrial contexts.

This study demonstrates the potential of using ECG-derived HRV features to physiologically assess the mental stress responses of inexperienced scaffolders under high-risk working conditions. From an academic perspective, the findings contribute to the growing body of knowledge on the relationships between workers’ physiological responses—particularly autonomic nervous system modulation reflected by HRV—and mental stress in construction settings. By focusing on inexperienced scaffolders operating under highly hazardous conditions, this study provides empirical evidence on how mental stress induced by high-risk tasks is manifested in physiological patterns. The identification of HRV features that are sensitive to stress-related autonomic changes offers theoretical support for future research on the physiological assessment of mental stress in occupational safety and human factor studies.

From an industry perspective, this study highlights the practical feasibility of HRV-based physiological assessment for monitoring workers’ mental stress on construction sites. The use of a single ECG sensor and a relatively small number of HRV features reduces the complexity of data acquisition and computational burden, which is advantageous for real-world deployment. Although the present study focused on inexperienced scaffolders, the proposed approach may also provide insights for monitoring mental stress among other novice workers engaged in high-risk tasks, such as working at height. In addition, the findings underscore the broader potential of ECG signals for assessing workers’ safety-related physiological states. Previous research has demonstrated the effectiveness of ECG-based measures in detecting physical fatigue (e.g., [49]), and the present study extends this application to mental stress assessment. Given the increasing availability of wearable ECG devices and their robustness to environmental noise, integrating ECG-based physiological monitoring into construction safety management systems may offer a promising direction for enhancing human-centric risk monitoring in construction environments.

One limitation of this study is that the participants were limited to young male novice workers (aged 19–23, with less than one year of experience). This is particularly important because growing evidence indicates that stress responses may differ between men and women, not only in subjective stress perception but also in physiological regulation, including autonomic nervous system activity reflected in HRV measures [50]. In addition, age and work experience have also been reported to influence HRV responses to mental stress [51]. Therefore, the findings may not be directly generalizable to female workers, older workers, or more experienced populations. In addition, mental stress in real construction environments may arise from multiple sources beyond working-at-height conditions, and the applicability of the identified HRV features and developed models to other stressors remains uncertain. From a methodological perspective, although this study adopted a widely used classical RR interval-based HRV computation and spectral analysis pipeline, such approaches may present limitations for short-term recordings, particularly for VLF components, due to the limited number of effective cardiac cycles within short analysis windows [52,53]. Moreover, although ECG was selected in this study because it provides direct measurement of cardiac electrical activity and more reliable R-peak detection under real-world conditions involving motion and environmental noise, it is less convenient for large-scale deployment than photoplethysmography (PPG), which is more commonly integrated into consumer wearable devices. In addition, although efforts were made to keep measurement times relatively consistent, strict control of sampling time across all participants was not always feasible in the field, and potential circadian influences on cortisol levels cannot be completely ruled out. Finally, the sample size was relatively small due to the field-based nature of the experiment and the practical constraints of collecting physiological data from construction workers performing high-risk tasks. Nevertheless, similar sample sizes have been commonly reported in related field studies (e.g., [12,13]), and the use of a within-subject design and leave-one-out cross-validation helped maximize the effective use of the available data and mitigate individual bias.

Future research should include larger and more diverse samples to improve the external validity of HRV-based stress assessment models. The applicability of the proposed approach across different types of occupational stressors also warrants further investigation. In addition, future studies may explore the integration of advanced HRV extraction methods, such as wavelet-based or adaptive approaches, to enhance robustness under short-term and noisy field conditions. Importantly, given the increasing availability of PPG-based wearable devices, future work should also investigate the feasibility and validity of PPG-derived HRV for mental stress assessment in construction settings.

## 6. Conclusions

This study investigated the potential of ECG-derived heart rate variability (HRV) features for physiologically assessing the mental stress responses of inexperienced construction workers operating under high-risk working conditions, specifically working at height. The results show that the HRV features have high accuracy in classifying mental stress levels. The prediction accuracy can be up to 92.50% for differentiating a low and a high level of mental stress, 85.00% for differentiating a low and a medium level of mental stress, and 87.50% for differentiating a medium and a high level of mental stress. The prediction models also showed good evaluation performance: low–medium (recall = 0.850, precision = 0.850, F1 = 0.850, AUC = 0.844), low–high (recall = 1.000, precision = 0. 870, F1 = 0. 930, AUC = 0.905), and medium–high (recall = 0.950, precision = 0. 826, F1 = 0. 884, AUC = 0.842). These findings suggest that ECG-derived HRV features provide a feasible and physiologically grounded approach for assessing construction workers’ mental stress in real job-site environments.

## Figures and Tables

**Table 1:** HRV features employed and their descriptions.

HRV Feature	Unit	Description (Equation)
Time domain features		
mRR	[ms]	The mean of RR intervals $(\frac{\sum_{i=1}^{N}(RR_{i})}{N}$)
SDRR	[ms]	The standard deviation of RR intervals $(\sqrt{\frac{\sum_{i=1}^{N}{\left(RR_{i}-mRR\right)}^{2}}{N-1}}$)
RMSSD	[ms]	The square root of the mean squared differences between successive RR intervals $(\sqrt{mean{\left((RR_{i+1}-RR_{i}\right)}^{2}}$)
pNN50	[%]	Number of interval differences of successive RR intervals greater than 50 ms ($\frac{count{\left(\left|RR_{i+1}-RR_{i}\right|\right)}_{>50ms}}{N-1}\times 100\%$)
mHR	[bpm]	Average heart rate
Frequency-domain features		
VLF	[ms^2^]	Absolute powers of very-low-frequency band (0–0.04 Hz)
LF	[ms^2^]	Absolute powers of low-frequency band (0.04–0.15 Hz)
HF	[ms^2^]	Absolute powers of high-frequency band (0.15–0.4 Hz)
TP	[ms^2^]	The total energy of RR intervals
LF/HF	[n.u.]	The ratio between LF and HF band powers
nLF	[n.u.]	Normalized low-frequency power
nHF	[n.u.]	Normalized high-frequency power
Non-linear features		
SD1	[ms]	The standard deviation for T direction in Poincaré plot
SD2	[ms]	The standard deviation for L direction in Poincaré plot

**Table 2:** Results of paired *t*-test between two stressful states.

HRV Features	Low (Mean ± SD)	Medium	High	Low–Medium		Low–High		Medium–High	
		(Mean ± SD)	(Mean ± SD)	t	*p*	t	*p*	t	*p*
mRR	748.40 ± 142.97	746.56 ± 139.84	738.45 ± 124.93	0.308	0.762	0.944	0.357	1.437	0.167
SDRR	24.23 ± 11.22	20.33 ± 8.54	21.15 ± 9.63	2.090	0.050	3.060	**0.006**	−0.921	0.369
RMSSD	22.71 ± 12.46	21.92 ± 12.37	20.58 ± 12.64	1.287	0.213	0.852	0.405	1.301	0.209
pNN50	6.39 ± 11.71	5.75 ± 12.85	5.67 ± 12.87	0.741	0.468	1.777	0.092	0.122	0.904
mHR	77.86 ± 11.86	80.10 ± 12.36	82.03 ± 13.00	−2.303	**0.033**	1.345	0.194	−3.256	**0.004**
VLF	40.11 ± 31.65	30.10 ± 23.12	20.26 ± 18.48	2.290	**0.034**	1.813	0.086	−1.345	0.194
LF	231.17 ± 30.07	108.00 ± 10.52	72.09 ± 56.94	2.360	**0.029**	2.319	**0.032**	−0.157	0.877
HF	289.97 ± 31.35	288.38 ± 34.38	251.51 ± 31.81	0.287	0.777	1.443	0.165	1.659	0.114
TP	568.31 ± 42.56	380.97 ± 39.07	362.49 ± 35.80	2.524	**0.021**	3.304	**0.007**	1.341	0.196
LF/HF	3.19 ± 0.54	1.33 ± 0.28	1.10 ± 0.21	2.154	**0.044**	2.027	0.057	0.472	0.642
nLF	41.22 ± 3.15	38.63 ± 2.59	33.13 ± 2.63	1.540	0.140	0.538	0.597	−1.563	0.135
nHF	55.77 ± 3.52	60.45 ± 2.66	65.11 ± 2.74	−1.679	0.106	−0.900	0.380	1.338	0.197
SD1	16.23 ± 8.92	15.65 ± 8.85	14.68 ± 9.03	1.309	0.206	1.014	0.323	0.789	0.440
SD2	29.52 ± 14.49	25.54 ± 11.29	23.71 ± 9.03	1.468	0.158	1.351	0.193	−1.185	0.251

**Table 3:** Summary of evaluations of classification models with the best accuracy.

	Applied HRV Features	Classification Accuracy	Model Evaluations
LM	mRR, SDRR, VLF, LF, TP, LF/HF	SVM, 85.00%	Recall = 0.850, Precision = 0.850, F1 = 0.850, AUC = 0.844
LH	SDRR, RMSSD, VLF, LF, TP, LF/HF	KNN, 92.50%	Recall = 1.000, Precision = 0.870, F1 = 0.930, AUC = 0.905
MH	mHR, mRR, RMSSD, VLF, HF, TP, nLF, nHF	KNN, 87.50%	Recall = 0.950, Precision = 0.826, F1 = 0.884, AUC = 0.842

## Data Availability

Some or all data, models, or code that support the findings of this study are available from the corresponding author upon reasonable request.
